# Household-Level Expenditure on Protective Measures Against Mosquitoes on the Island of La Réunion, France

**DOI:** 10.1371/journal.pntd.0002609

**Published:** 2014-01-02

**Authors:** Josselin Thuilliez, Claire Bellia, Jean-Sébastien Dehecq, Olivier Reilhes

**Affiliations:** 1 CES-CNRS, Université Paris 1, Panthéon-Sorbonne, Centre d'économie de la Sorbonne, Maison des Sciences Economiques, Paris, France; 2 Institut de Recherche pour le Développement (IRD), Parc Technologique Universitaire, BP172 - 97492 Sainte Clotilde Cedex, La Réunion, France; 3 ARS Océan Indien, Saint-Denis, France; Centers for Disease Control and Prevention, Puerto Rico, United States of America

## Abstract

**Background:**

For decades La Réunion has experienced a number of epidemics that have resulted in efforts to control the density of *Aedes* species on this Island. This study was conducted to assess household-level expenditure on protective measures against mosquito nuisance on the Island of La Réunion in 2012.

**Methodology/Principal Findings:**

Data was collected during a cross-sectional survey of 1024 households and used to determine the relationship between the use of chemically-based protective measures and subjective and objective indicators of the density of *Aedes albopictus*. The average household expenditure in July 2012 was USD 9.86 and the total household-level expenditure over a one-year period was extrapolated to USD 28.05million (range: USD 25.58 million to USD 30.76 million). Much of this money was spent on measures thought to be relatively ineffective against *Aedes* mosquitoes. Expenditure on protective measures was not influenced by the level of knowledge on mosquitoes or by the visual nuisance they generated at home, but rather by the perception of risk related to a future epidemic of chikungunya and socioeconomic factors. Most importantly, household spending on protective measures was found to be influenced by a measure of zone-level mosquito density (the Breteau index), but not by objective indicators of the presence of mosquitoes within or around the house.

**Conclusions/Significance:**

Household-level expenditure on chemically-based protective measures is high when compared to the investment made by public entities to achieve vector control, and it is differentially influenced by subjective and objective measures of mosquito density. The current situation could be improved, firstly by ensuring that the public is well-informed about mosquitoes and the effectiveness of various protective measures, and secondly by implementing interventions that could either complement current vector-control strategies and improve their effectiveness on a country-level, or that would steer the population toward the appropriate behaviours.

## Author Summary

The French Ministry of Health has, for decades, dedicated numerous resources to control mosquito density on the Island of La Réunion. These efforts were strengthened following an outbreak of chikungunya, a virus transmitted by *Aedes* mosquitoes, in 2005–2006. In order to understand how public perception and behaviour is affected by this vector, a study was undertaken in 2012. Public behaviour was assessed using estimates of household expenditure on protective measures against mosquitoes. Information was gathered using a survey administered to 1024 households on the Island. Knowledge about mosquitoes was found to be poor across the sample, while perceptions of a risk from epidemics were high. The threat of a chikungunya epidemic was found to be associated with increased expenditure on protective measures, as was a zone-level measure of mosquito density, the Breateau Index. The most important finding is that overall household expenditure due to mosquitoes over a one-year period is USD 28.05 million, rather high when compared to the public service investment. Future vector-control in La Réunion needs to ensure that public health messages are understood by the population and that interventions are implemented that promote appropriate behaviours and reduce current spending at the household-level on protective measures.

## Introduction

*Aedes albopictus*, commonly known as the Asian tiger mosquito, is an anthropophilic, daytime biting species that rapidly establishes itself in new urban areas owing to its propensity to breed in both artificial and natural containers of stagnant water [1]. The tiger mosquito is particularly threatening owing to its potential for transmitting a wide range of arboviruses, including dengue and chikungunya viruses, yellow fever virus, and several other types of encephalitides [2], [3], [4], [5], [6].

La Réunion is one of the places in the world that has experienced a number of epidemics as a result of the favourable conditions it provides for mosquito species to thrive. Past outbreaks of malaria and dengue prompted authorities on the Island to implement strategies to control mosquito density. Following the resurgence of dengue in 2004, the local vector control services, referred to as the ‘Services de Lutte Antivectorielle’, or LAV, started developing a control strategy targeted at urban vectors, primarily *Aedes albopictus* [7], [8], [9]. The major chikungunya outbreak that swept through La Réunion in 2005–2006 created even stronger motivation for authorities to set up entomologic surveillance of *Aedes albopictus* in all urban areas. This surveillance effort continues today through monitoring of traditional stegomyia indices of immature stages (i.e. Container Index, House Index, Breteau Index) as are used in other control programmes [10]. The house index is defined as the percentage of houses infested by larvae and/or pupae. The container index is defined as the percentage of water-holding containers with active immature stages of mosquitoes. The Breteau index is defined as the number of positive containers per 100 houses, a positive container being one that contains larval and/or pupal stages of mosquito. The Breteau index is being used as a measure of zone-level vector density in this analysis (see method section).

*Aedes albopictus* remains the main target of the work of the LAV, a service which is provided by the Regional Health Agency (Agence Régionale de la Santé, or ARS) in La Réunion. The vector-control strategy integrates five core activities: vector surveillance, environmental, mechanical, and chemical control, and public health education campaigns [11]. Another aspect of the work of the vector control services is the early detection and treatment of cases of arboviral infection to prevent the spread of new epidemics. For the most part, the day-to-day activities of LAV officers involve education and promotion of vector-control at the household-level. These officers routinely visit households in La Réunion and provide education to families on the importance of eliminating sources of stagnant water around the house, such as emptying water from pots and saucerss placed under potted plants.

Given the investment of both financial and human resources toward the control of *Aedes albopictus* in La Réunion, a study was proposed to assess the population's perceptions and behaviour related to mosquito nuisance, and identify whether the current strategy could be improved or enhanced through new vector-control measures or interventions. For this study, insight into household-level behaviour was gained using estimations of expenditure on protective measures against mosquitoes. The objective was to determine whether spending at the household-level is influenced by subjective or objective exposure to *Aedes* mosquitoes on the Island of La Réunion, and whether this level of expenditure warrants action by public authorities to improve current vector-control strategies.

## Methods

### Study site

This study was performed in urban areas of La Réunion. The Island is divided into 4 geographic sectors (North/South/East/West), 24 municipalities and 273 neighbourhoods. These neighbourhoods are divided into 960 zones used by the ARS for *Aedes albopictus* surveillance and control. These homogenous zones are defined according to urban planning and environmental criteria, they extend over 275 km^2^ or 11% of the Island, and mostly cover urban areas. According to the National Institute of Statistics and Economic Studies (or INSEE) in France, an urban area is defined as an agglomeration of more than 2000 residents where no dwelling is separated from the next closest dwelling by >200 metres [12].

Home-owners generally allow LAV officers of the ARS to enter into their private dwellings to carry out routine vector-control activities. We commenced our sampling technique by selecting all zones controlled by the ARS on a minimum of three occasions between 2007 and 2011.We used zones located near the coast (less than 500 metres in altitude) where the presence of mosquitoes from one year to the next is most likely to persist and to ensure a relative homogeneity in environmental factors. Next, we focused on zones that showed relative stability in mosquito density between 2007 and 2011and we classified these as either negative or positive zones using criteria based on the Breteau Index (i.e. the number of positive containers per 100 houses). Using the historical data gathered over this 5-year period, we defined a negative zone as one that had a value for the Breteau Index at LAV routine visits that was lower than 50% of the average Breteau Index value during the same month and year in all zones. A positive zone was defined as having a value for the Breteau Index during LAV routine visits that was higher than 50% of the average Breteau Index value during the same month and year in all zones. A zone was kept for inclusion in the study if it was classified as positive or negative a majority of times during all LAV routine visits (e.g. classified as ‘positive’ twice during three routine visits conducted between 2007 and 2011). This was done to ensure a degree of stability in the classification of zones through time. A total of 184 zones, 68 positive zones and 116 negative zones, were identified using this methodology.

### Participants and procedures

Being that face-to-face interviews in 184 zones were not feasible, a two-stage cluster random sample was drawn from this first selection. In the first stage of this two-stage sampling technique, a random sample of 26 zones (13 positive and 13 negative) was drawn taking into account the geographic distribution of the population on the Island in the four sectors (North/South/East/West). Next, households were randomly selected to achieve a fixed sample of 40 households per zone. The selection of households was undertaken while LAV officers were in the field. All households were randomly chosen and surveys were conducted in locations routinely checked by vector control officers. For each zone, the officers were asked to interview residents living in alternate households. The households were selected in this way while walking through the zone. The LAV officers commenced the survey starting at the four corners of the zone and walked in varying directions that were also chosen at random. The percentage of absentees and refusals varied considerably between zones; the average percentage of absentees was 36% (range of 5% to 50%), and the average percentage of refusals was 12% (range of 4% to 24%). The reason most often quoted for refusing to participate in the survey was a lack of time to respond to the questionnaire. For each selected household, the LAV officers conducted both a face-to-face interview that was addressed to the head of the household as well as an observational survey of the outside of the dwelling itself. The data collected were validated by comparing key characteristics of our sample to information provided in the latest census (e.g. number of household members by age of the head of household, level of education of the head of household, socioeconomic status). No significant differences were found for these key characteristics, an indicator that our sample was representative of the population in La Réunion. Conversely, a significant difference for the Breteau Index measured during the month of interviews (i.e. July 2012) was found between the positive and negative zones in the sample (Mann-Whitney test p-value<0.001). This finding confirms that the survey provides a rather accurate picture of the long-term average density of *Aedes albopictus* in the selected zones (i.e. our sampling technique resulted in the selection of zones that retained their characteristic classification of vector density through time).

### Questionnaire and measures

Questions were derived from existing literature on protective behaviours against mosquitoes [13], [14], [15], [16], [17], [18]. Due to the circulation of dengue virus at the time of the study and an epidemic alert level up to 2B issued by the ARS during the month of April 2012, questions on the risk and perception of dengue were also integrated into the questionnaire. A pilot study was launched in June 2012 to test the validity of the questionnaire. The main study was carried out in July 2012, and was conducted according to the rules established by the National Data Protection Authority. Informed consent to answer the 40-minute questionnaire and to allow a LAV officer to conduct an observational survey of the residence was obtained verbally from all participants at the beginning of the interview. Translators were used when necessary.

#### Household socio-economic data

A relative index of household socioeconomic status (SES) was derived based on dichotomous variables (durable goods and assets such as TV sets, cars, housing infrastructure, etc) using principal components analysis (PCA). Information on ownership of these assets was used to generate an index of long-run wealth which is thought to explain the maximum variance and covariance in the asset variables [19]. For many economists, household income or consumption expenditure are the indicators of choice for socioeconomic status. However, the pilot study as well as discussions with locals showed that it would be difficult to collect such data through direct questioning, particularly since LAV officers are government employees. To overcome this and ensure that the questionnaire does not become too long, it was decided to use information on assets owned by household members and characteristics of the house to develop an indicator of SES. This is a method also described by Filmer and Pritchett [18]. The wealth index derived using asset variables has been shown to be a good proxy for long-run economic status.

To confirm whether this wealth index could provide a good picture of the SES of different households we compared our data with that provided by other sources. In the first instance, our survey showed that the East of the Island was significantly poorer than the rest of La Réunion which is in accordance with previous results on unemployment and the economic situation in La Réunion. When comparing ownership of specific assets (e.g. car, washing machine) the data collected in this study were comparable to figures provided in previous censuses. For instance the percentage of households having at least one car was 69.9% in the 2009 census compared to 71.6% in our survey in 2012. Age, gender, level of education of the head of household, work status, and information on the size of the household were also collected so as to be included as confounders in the regression analysis. The percentage of heads of households that were female was 63.67%.

#### Subjective exposure to mosquitoes

To assess subjective exposure, two variables were used; participants were asked if they had mosquitoes in their dwellings or their direct environment (dichotomous variable), and how frequently they were bitten by mosquitoes while at home (ordinal variable). Subjective exposure, as opposed to objective exposure, is based on what an individual perceives or what one considers themselves to be exposed to, and such a measure is therefore inherently variable from one individual to the next, and open to personal interpretation. Several questions that assessed knowledge on mosquitoes and mosquito behaviour, as well as the infectious diseases they transmit were also included in the questionnaire.

#### Objective exposure to *Aedes albopictus*

Part of the questionnaire was dedicated to direct observation of the garden and direct surroundings for the presence of stagnant water and breeding sites for mosquitoes. Only the indices of breeding sites for *Aedes albopictus* were measured during this survey. LAV officers are sensitised to the preference of different mosquito species in terms of breeding habitat and routinely collect information on *Aedes albopictus* during their door-to-door activities. The officers counted the number of sources of stagnant water (whether in natural or artificial containers) that (1) did not contain any *Aedes* mosquito larvae or pupae, (2) that contained only larvae but no pupae, and (3) that contained both larvae and pupae. Sites (2) and (3) are referred to as ‘positive breeding sites’ for the purposes of this study. The number of such sites as well as the typology, e.g. vase, gutter, abandoned car tyre, and saucers placed under pots, were noted on a standard document provided to the LAV officers.

The number of positive *Aedes* breeding sites was used as a measure of *household-level exposure* to mosquitoes (i.e. the number of containers of stagnant water containing *Aedes* pupae and/or larvae around the house). To capture the level of exposure to mosquitoes across the entire zone (i.e. a measure of *zone-level exposure*), the Breteau Index was used (i.e. the number of positive *Aedes* containers per 100 houses in a zone). In both cases, the indices are referred to as ‘objective’ measures of exposure because the measurement is made using counts and is therefore not subject to interpretation by different individuals.

#### Protective behaviours and related expenditure

Respondents of the questionnaire were asked to provide a list of chemical, physical, and ecological measures used to protect themselves from mosquito-related nuisance during the month of the interview. This question was completed and cross-validated using a closed question that asked the interviewee to select from a list of specific products or measures. In order to assess the expenditure on protective measures used by the study sample we started by compiling a list of all brands of products recommended by the HAS (Haute Autorité de la Santé, or the French Authority of Health) and judged effective by this Authority. This list, which is available to the public, includes information on the type of products, the active substance, and brands available for a number of protective measures [20]. A list of other products commonly cited by respondents during the interviews but judged less effective against the prevention of chikungunya and dengue by the HAS (e.g. mosquito coils, fans, air conditioning, and treated/untreated mosquito nets for adults) was added to this first list of products in order to capture all measures used in households across La Réunion. To our knowledge, there are relatively few papers evaluating the efficacy and cost-effectiveness of a broad range of protective measures against *Aedes albopictus* in non-laboratory conditions. Next, the exact price of different brands of each product was collected from up to five different retailers and from e-commerce websites. This was done to ensure comprehensiveness in determining the average price of each product [21]. About 250 products were identified and listed, with approximately 1250 prices collected in total. Weighted averages (taking into account intra-brand variability) were then calculated for each category of product (Table 1). Prices of products with a lifespan greater than or equal to one year (such as air conditioning, mosquito nets, and fans) were discounted using a 5% annual rate following standard practice. Household expenditure is expressed in USD (the Average Bid rate for the week of Monday, Jul 16, 2012 to Sunday, Jul 22, 2012 was used as the reference being that this week corresponds to the middle date of the survey; EUR 1 = USD 1.224) [22].

**Table 1:** Average price estimates of product categories and associated standard errors (in ascending order of price).

Product	Average price (in USD)	SD	Median Price (in USD)	Proportion of households using each of the major control products
Mosquito coils	4.71	2.59	5.94	69.04
Insect/mosquito repellent sprays for the house	4.99	3.20	4.55	52.73
Anti-mosquito window stickers	5.07	2.39	5.51	0.10
Tablets	5.12	1.88	4.16	0.00
Non-electric diffusers with or without recharge	5.20	1.18	4.64	38.09
Rechargeable electric vaporizers/diffusers	5.84	2.70	4.46	19.43
Repellent bracelets	7.85	1.85	6.64	2.25
Anti-mosquito patch	7.87	3.33	7.10	0.10
Essential oils	8.50	2.82	8.20	22.85
Candles	8.93	5.56	8.84	0.58
Plants: citronella, geranium	9.15	5.31	8.77	18.55
Citronella-based sprays for the house	9.53	4.84	11.87	0.00
Anti-mosquito body sprays/creams	10.19	4.05	8.51	36.82
Rackets and swatters (electric or not)	10.59	4.51	10.47	7.23
Impregnated clothing	10.97	3.90	9.39	0.00
Insecticide powder	14.28	3.02	14.02	0.39
Ultra-sound devices	15.23	4.61	13.76	0.29
Electric traps	18.12	13.98	15.11	0.00
Treated and untreated mosquito nets	38.62	9.20	43.40	14.26
Fans	84.61	72.68	48.85	19.73
Anti-insect/anti-mosquito lamps	156.42	163.97	132.11	0.10
Air-conditioning	1100.27	570.54	1036.90	8.11

**Figure 1:**
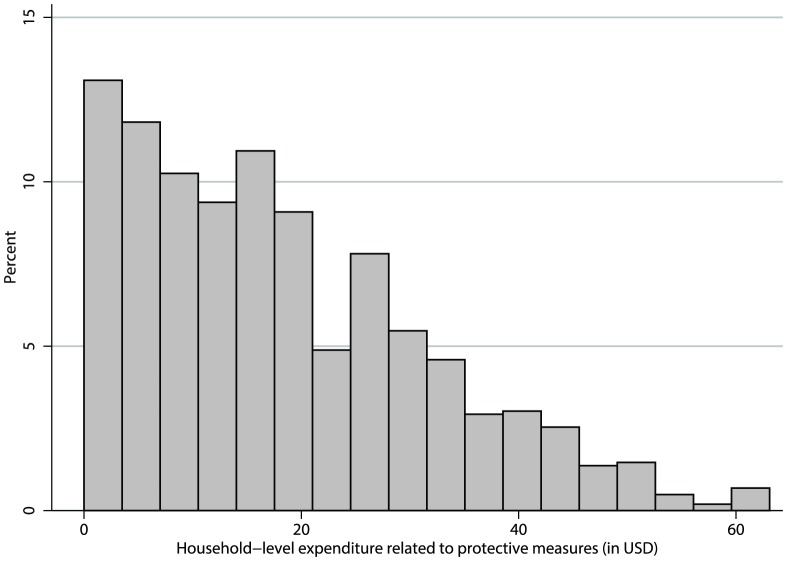
Distribution of household-level expenditure related to protective measures.

Typically, the distribution of household direct expenditure related to chemical, physical, and ecological protective measures, is positively skewed (Figure 1). We minimised the influence of high household-level expenditure on the regression analysis by applying the inverse hyperbolic sine (IHS) transformation; sinh^−1^(*y*) = log(*y*+(*y* ^2^+l)^1/2^) [23]. The sinh transformation was then used to extrapolate adjusted measures of household direct expenditure (sinh(*x*) = ½ (e*^x^*−e^−x^)) and provide estimates in USD from the regression model.

### Data analysis

Analysis was performed using STATA/SE v11.0 (StatCorp, College Station, TX). Descriptive information on behaviour, knowledge, and perceptions related to mosquitoes in our study sample was extracted using univariate and bivariate analyses. However, extrapolating from a simple average expenditure per household, as well as the relationship between expenditure per household and Breteau index, could be confounded by other household- or zone-level cofactors (presence of mosquitoes at home, wealth index, education amongst others), resulting in biased estimates at the Island level. In light of this we decided to analyse the influence of household- and zone–level characteristics on expenditure on protective measures (i.e. direct expenditure at the household level) using multivariate regression analysis. Simple generalised linear regression analysis was first performed using household-level expenditure on protective measures as the outcome variable. All factors significantly related to household-level expenditure were then entered into the final multiple regression model, using a random effects model. Provided that random effects are uncorrelated with the fixed predictors in the model, a random effects model is preferable, as it allows for the consideration of both household- and zone-level characteristics in a single model [24]. As the number of zones is relatively low (26), we didn't perform a mixed multilevel analysis, following Scherbaum et al recommendations (in which case a minimum of 30 level 2 zones is recommended to perform multilevel analysis) [25]. Lastly, univariate sensitivity analysis was conducted to determine how sensitive our estimates of household expenditure were to variation in input parameters, including perceived risk of a new epidemic of chikungunya, age, wealth, and education. Analysis was performed on wealth quintiles distribution across the population (from 100% in poorest category to 100% in richest category), educational levels (from 100% in the primary education category to 100% in college and higher category), age (20 to 99 years old), and perceived risk of a new epidemic of chikungunya (from all people considering that there is a low risk to 100% considering that there is a high risk). We also tested the influence of excluding air-conditioning and fans from the expenditure calculation. Results are displayed graphically using a tornado diagram.

## Results

### Sample characteristics

**Figure 2:**
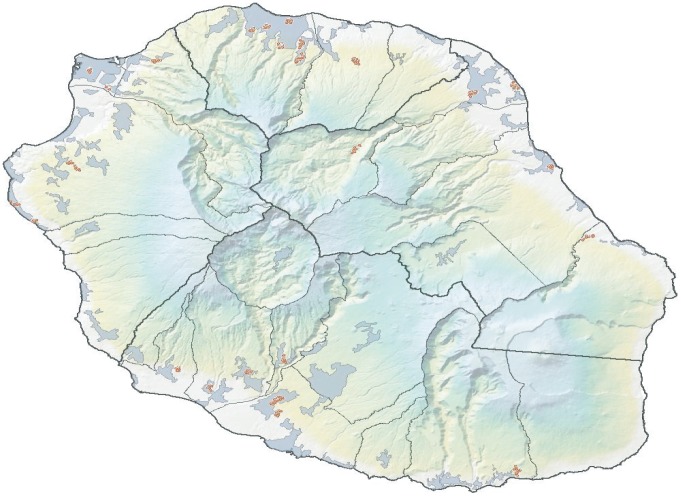
Location of sampled zones in La Réunion. Red dots provide the approximate location of zones of households interviewed. Urban areas are shaded in a darker grey.

A total of 1024 households in La Réunion were interviewed over the month of July 2012. Figure 2 provides the location of zones interviewed on the Island and Table 2 provides descriptive statistics of the main factors included in the multivariate analysis.

**Table 2:** Descriptive statistics.

Variables			
**Dependant variable**			
	Inverse hyperbolic sine transformation of household expenditure (in USD) - mean (sd)		3.06 (1.33)
**Subjective exposure to mosquitoes**			
	Presence of mosquitoes at home (yes/no) - N° (%)		792 (77.34)
	Frequency of mosquito bites in the last 7 days - N° (%)	Never	586 (57.23)
		Seldom	372 (36.33)
		Sometimes	50 (4.88)
		Often	16 (1.56)
**Objective exposure to Aedes albopictus**			
	Number of positive breeding sites (at household level) - mean (sd)		0.422 (1.14)
	Breteau Index (at cluster level) - mean (sd)		42.28 (21.96)
**Household characteristics**			
	Wealth Index - N° (%)	Quintile 1 (poorest)	204 (19.92)
		Quintile 2	205 (20.02)
		Quintile 3	205 (20.02)
		Quintile 4	205 (20.02)
		Quintile 5	205 (20.02)
	Education - N° (%)	None	64 (6.25)
		Primary school	233 (22.75)
		Secondary school	235 (22.95)
		High school	342 (33.40)
		College and higher	150 (14.65)
	Age of respondent - mean (sd)		52.36 (16.55)
	Gender of respondent is female - N° (%)		652 (63.67)
	Number of children in the household - mean (sd)		0.79 (1.15)
**Knowledge on mosquitoes**			
	Female is biting gender - N° (%)		394 (38.48)
	Average distance travelled by a mosquito is less than 500 meters - N° (%)		468 (45.70)
	Diseases transmitted by mosquitoes (score between 0 and 5) - mean (sd)		3.73 (1.23)
**Perceived risk of a new epidemic**			
	Chikungunya - N° (%)	No risk	73 (7.13)
		Low risk	303 (29.59)
		Reasonable risk	324 (31.64)
		High risk	165 (16.11)
		Don't know	159 (15.53)
	Dengue - N° (%)	No risk	79 (7.71)
		Low risk	256 (25.00)
		Reasonable risk	323 (31.54)
		High risk	200 (19.53)
		Don't know	166 (16.21)
**Perceived effectiveness of personal control measures - N° (%)**		No	55 (5.37)
		Not really effective	247 (24.12)
		Effective	546 (53.32)
		Really effective	118 (11.52)
		Don't know	58 (5.66)

### Mosquito-related knowledge

Only about 40% percent of respondents could identify the female as the biting gender in mosquito species. When asked to identify diseases transmitted by mosquitoes, the mean score for respondents in the study sample was of 3.73 correct answers out of 5. Not surprisingly, 92% of interviewees knew that chikungunya is a disease transmitted by mosquitoes, and another 78% answered correctly for malaria. More than a quarter of the respondents answered incorrectly that influenza is a disease transmitted by mosquitoes.

### Perceived nuisance

Most respondents declared that mosquitoes were present in their homes (77%). However, when asked about the frequency of mosquito bites over the last 7 days, 93% claimed that they were seldom or never bitten by mosquitoes. In spite of the low level of mosquito bites, 90% of interviewees considered mosquitoes a nuisance and 63% stated that these insects were of no particular use. When asked why mosquitoes were a nuisance, more than 80% replied that this was due to their role in transmitting diseases, 80% stated that mosquito bites and itching were important reasons, and 65% stated that it was due to the noise they created.

### Perceived risk

When questioned about the risk of epidemics, 47.7% of interviewees perceived that the risk of a new outbreak of chikungunya was reasonable or high. On the other hand, the risk of a dengue epidemic was perceived as being reasonable or high by 52% of respondents. This higher perceived risk of dengue could be explained by the increased attention given to this disease during the time of the study when a small epidemic of dengue was unfolding across the Island. Interestingly, however, when an open question was posed about which diseases could be transmitted by mosquitoes, only 20% of respondents spontaneously quoted “dengue”.

### Perception of vector-control efforts

With respect to overall perception of vector control efforts, approximately 70% of respondents were confident that it is possible to reduce the number of mosquitoes. However, 20% insisted that nothing could be done in this regard. More than 75% of interviewees think that science could make further advances in the field of vector control. In terms of the acceptability of different measures to control mosquito numbers, 97% find the elimination of stagnant water acceptable, 82% feel that measures to repel mosquitoes are acceptable, and 74% accept techniques that prevent the reproduction of mosquitoes. Insecticide spraying is deemed acceptable by 65% of the study sample.

### Individual-level protective behaviour

When asked whether they eliminate sources of stagnant water in and around their households, 97% of respondents declared that they did this. The frequency of this behaviour varied, however, with 17% claiming that they would eliminate these sources at least once per day, 45% declaring that they did this a few times per week, 11% stating that they did it a few times per month, and about 2% stating once per year. About 23% of the study sample claimed that they had definitively eliminated potential sources of stagnant water by removing empty containers or other potential recipients from their surroundings.

Among the various measures listed in the questionnaire, mosquito coils emerged as the most commonly used protective measure in this study, with 69% quoting that they used this measure at the time of the study. Insecticide/mosquito repellent sprays for the house (53%), non-electric diffusers (38%), and repellent creams and sprays applied to the skin (36%) were less frequently used by the sample during this period. Although use of these measures persists, about 50% of respondents stated that they consider them to be either reasonably or very dangerous to one's health, and another 24% felt that these products were not really effective. According to the health recommendations for travellers [20], mosquito coils are amongst the least effective measures to repel *Aedes* mosquitoes, intra-domiciliary insecticides and electric diffusers were found to have limited and weak effectiveness, and repellent creams and sprays applied to the skin were judged to be of stronger effectiveness. Therefore, the utilisation pattern of protective measures in this study population appears to be directly inverse to the recommendations of the health authorities [20] in terms of product effectiveness.

The lists provided by respondents of the measures they use to protect themselves against mosquitoes were used to determine the average household-level expenditure on these measures during the study period. This average expenditure per household was estimated to be USD 18.09 during the month of July 2012 and the median expenditure was estimated at USD 15.54. We tested the robustness of these household-level expenditure estimates by verifying whether these correspond with the results of a direct and closed question on household expenditure in the questionnaire. Respondents were asked to specify whether they judged their monthly spend on protective measures against mosquitoes to be less than EUR 10 (USD 12.24), between EUR 10 (USD 12.24) and EUR 20 (USD 24.48), EUR 21 (USD 25.70) to EUR 40 (USD 48.96), or more than EUR 40 (USD 48.96). Just over 60% of respondents declared spending between USD 12.24 and USD 24.48 per month on protective measures against mosquitoes. Taking the middle point of the range of each category, we found an average expenditure of USD 13.60, when using the results of this direct and closed question. A direct declaration of monthly expenditure (i.e. a categorical variable) is judged to be less reliable and accurate compared to the estimation of expenditure based on a list of used products (continuous variable) which is why the latter, i.e. the estimates of household-level expenditure, have been used as the dependent variable in the regression analysis.

### Relationship between potential predictors and household-level expenditure on protective measures against mosquito bites

Table 3 summarises the findings of the multivariate analysis that shows household-level expenditure on protective measures against mosquitoes as the dependent variable. Wealth quintiles, age (*p* = 0.039), and an educational level above a college degree (*p* = 0.087), were found to be positively and significantly associated (at the 10% level) with household-level expenditure on protective measures. Gender of the head of the household was not found to influence expenditure.

**Table 3:** Multivariate regression analysis (random effects model).

			Dependent variable is inverse hyperbolic sine transformation (IHS) of household expenditure (in USD).				
			Coefficient	95% Conf. Interval			p Value
**Subjective exposure to mosquitoes**							
	Presence of mosquitoes at home (yes/no) - Ref is No		0.135	−0.071	-	0.342	0.200
	Frequency of Mosquito bites in the last 7 days	Never - Ref					
		Seldom	0.198	0.016	-	0.380	0.033
		Sometimes	0.192	−0.195	-	0.579	0.331
		Often	0.588	−0.067	-	1.242	0.078
**Objective exposure to ** ***Aedes albopictus***							
	Number of positive breeding sites (at household level)		0.034	−0.037	-	0.106	0.348
	Breteau Index (at cluster level)		0.006	0.002	-	0.009	0.004
**Household characteristics**							
	Wealth Index	Quintile 1 (poorest) - Ref					
		Quintile 2	0.230	−0.027	-	0.487	0.079
		Quintile 3	0.352	0.081	-	0.624	0.011
		Quintile 4	0.230	−0.052	-	0.512	0.110
		Quintile 5	0.447	0.153	-	0.740	0.003
	Education	None - Ref					
		Primary school	0.149	−0.215	-	0.514	0.422
		Secondary school	0.281	−0.119	-	0.681	0.169
		High school	0.253	−0.169	-	0.674	0.240
		College and higher	0.413	−0.059	-	0.886	0.087
	Age of respondent		0.007	0.000	-	0.014	0.039
	Gender of respondent		0.088	−0.079	-	0.255	0.301
	Number of children in the household		0.018	−0.063	-	0.098	0.670
**Knowledge on mosquitoes**							
	Female is biting gender		−0.103	−0.281	-	0.076	0.261
	Average distance travelled by a mosquito is less than 500 meters		0.084	−0.077	-	0.245	0.308
	Diseases transmitted by mosquitoes		0.057	−0.019	-	0.133	0.141
**Perceived risk of a new epidemic**							
	Chikungunya	No risk - Ref					
		Low risk	0.531	0.187	-	0.874	0.002
		Reasonable risk	0.602	0.253	-	0.951	0.001
		High risk	0.619	0.229	-	1.009	0.002
		Don't know	0.368	−0.011	-	0.747	0.057
	Dengue	No risk - Ref					
		Low risk	−0.066	−0.410	-	0.277	0.705
		Reasonable risk	0.026	−0.318	-	0.369	0.884
		High risk	−0.023	−0.393	-	0.347	0.903
		Don't know	−0.067	−0.429	-	0.296	0.719
**Perceived effectiveness of personal control measures**		No - Ref					
		Not really effective	0.285	−0.095	-	0.665	0.141
		Effective	0.427	0.064	-	0.790	0.021
		Really effective	0.158	−0.262	-	0.577	0.461
		Don't know	−0.252	−0.738	-	0.235	0.311
**Intercept**			0.707	−0.110	-	1.525	0.090

Knowledge on diseases transmitted by mosquitoes is shown to positively affect household expenditure on protective measures but the coefficient of this variable just misses the statistically significant threshold of 10% (*p* = 0.141). Correct knowledge of the biting gender of the mosquito species or the distance travelled by mosquitoes had no significant impact on expenditure on protective measures. In terms of perceived risk of a potential epidemic, the perceived threat of another chikungunya outbreak was found to be significantly associated with expenditure on protective measures. The potential threat of a dengue epidemic, however, did not have the same effect. Perception of the effectiveness of protective measures was found to influence household expenditure on these items only for those who stated that these measures are ‘effective’ (*p* = 0.021).

### Relationship between subjective and objective exposure to mosquitoes and household-level expenditure on protective measures

The regression analysis shows that while the declaration of having mosquitoes in one's household does not appear to influence expenditure on protective measures, an estimation of the frequency of mosquito bites given by respondents, on the other hand, has a significant and positive relationship with expenditure on products. There is a clear relationship between the perceived biting frequency (a measure of subjective exposure to mosquitoes) and the degree of spending on protective measures (the response to this exposure).

In terms of objective evidence of mosquito density, the number of positive *Aedes* breeding sites measured in and around the household does not appear to influence expenditure, and it is in fact the vector density for the entire zone (measured using the Breteau index), that has a positive impact on household spending on protective measures (*p* = 0.004).

### Extrapolating household expenditure for La Réunion from July 2011 to July 2012

**Figure 3:**
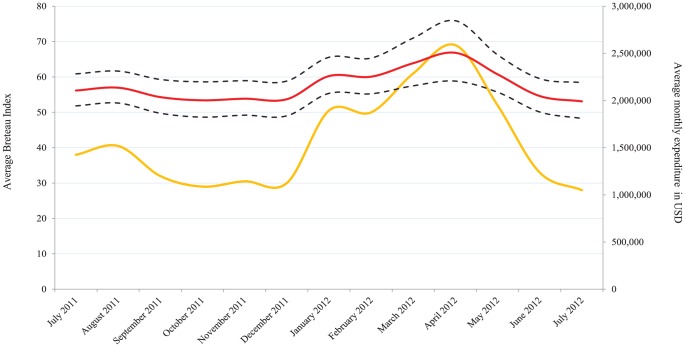
Variation of the estimated expenditure on protective measures against mosquitoes in La Réunion (red line and associated 95% CI in black dotted lines) with changes in the average Breateau Index (yellow line) over a one-year period.

Assuming that the positive relationship between the Breteau Index and household spending exists for all seasons of the year, it was possible to extrapolate the results of this study to make household expenditure estimations for the months between July 2011 and July 2012. A prerequisite for this extrapolation was data on the seasonal variation of the Breteau Index over this one-year time-frame, which were obtained using ARS records of monthly-measured Breteau index (publicly available on a monthly basis on the ARS website [26]). When asked about mosquito nuisance 72% of participants claimed to be affected by this mostly during the Austral summer (November to April), and another 42% declared that their use of protective measures would increase during the year, a plausible finding being that this study was conducted during the month of July. The indices recorded by the ARS were compared with the results from our regression analysis (provided in Table 3). We used predictions of the model at fixed values of ARS-measured Breteau index and averaged over the remaining significant covariates to estimate an annual global expenditure for households on the Island. Results for both the average expenditure per household and for all households in Réunion are given in Figure 3 and Table 4. In this study, the definition of a household has been limited to persons living within houses. The total number of households in La Réunion in 2009 was 284,391, as measured by the National Institute of Statistics and Economic Studies in France (INSEE). Among them, INSEE estimates that around 71% reside in houses. This corresponds to a total of 201,917 households (as defined in this study) on the Island of La Réunion.

**Table 4:** Extrapolating household expenditure for La Réunion.

Month	Average Breteau index (source: ARS)	Predicted IHS expenditure per household	IHS Lower bound per Household	IHS Upper bound per Household	Corresponding Average expenditure per Household in USD	Corresponding Lower Bound per Household in USD	Corresponding Upper Bound per Household in USD	Estimated expenditure for La Réunion in USD (201,917 households)	Expenditure Lower Bound for La Réunion (201,917 households)	Expenditure Upper Bound for La Réunion (201,917 households)
July 2011	38.00	3.04	2.96	3.12	10.43	9.63	11.31	2,106,626	1,943,586	2,283,206
August 2011	40.50	3.05	2.98	3.13	10.58	9.77	11.45	2,136,373	1,973,692	2,312,334
September 2011	32.00	3.01	2.92	3.09	10.09	9.24	11.01	2,036,895	1,865,500	2,223,869
October 2011	29.00	2.99	2.90	3.08	9.92	9.03	10.89	2,002,892	1,824,266	2,198,815
November 2011	30.50	3.00	2.91	3.09	10.00	9.14	10.95	2,019,824	1,845,015	2,211,013
December 2011	30.00	3.00	2.90	3.09	9.98	9.10	10.93	2,014,164	1,838,125	2,206,878
January 2012	50.50	3.11	3.03	3.19	11.19	10.28	12.18	2,259,584	2,076,110	2,459,133
February 2012	50.00	3.11	3.02	3.19	11.16	10.26	12.14	2,253,259	2,071,672	2,450,628
March 2012	61.00	3.17	3.06	3.27	11.87	10.68	13.19	2,396,548	2,156,490	2,663,120
April 2012	69.00	3.21	3.09	3.34	12.41	10.93	14.10	2,506,406	2,206,511	2,846,764
May 2012	52.00	3.12	3.03	3.21	11.29	10.35	12.31	2,278,666	2,089,040	2,485,354
June 2012	33.00	3.01	2.93	3.10	10.14	9.31	11.06	2,048,357	1,878,981	2,232,839
July 2012	28.00	2.98	2.89	3.08	9.86	8.97	10.85	1,991,684	1,810,313	2,191,021
Total Annual expenditure	-	-	-	-	138.924	126.682	152.364	28,051,277	25,579,300	30,764,977

The analysis shows that the predicted average household expenditure for July 2012 is USD 9.86, which is a better estimate of household expenditure than that derived from the list of used products as explained in the descriptive statistics section. This figure is also more accurate because it takes into account confounders included in the regression analysis. The projected total annual expenditure per household was estimated to be USD 138.92 (when using the survey definition of a household). If we include all households on the Island and assume that residents living in dwellings other than houses have no mosquito-related expenditure (i.e. taking a conservative approach), the total annual expenditure per household is USD 98.63 (the lower bound estimate of the total annual expenditure per household).

Overall, the amount spent by 201,917 households in Réunion from July 2011 to July 2012 on personal protective measures is estimated to be USD 28.05 million (95% CI of USD 25.58 million to USD 30.76 million). By comparison, other authors have found that the chikungunya epidemic resulted in medical expenses of up to EUR 43.9 million (USD 53.74 million) [27]. The annual Gross Domestic Product (GDP) of La Réunion was estimated to be EUR 14.42 billion (USD 17.65 billion) in 2009. Hence, the household-level expenditure related to protective measures in La Réunion amounts to 0.15% of its annual GDP in 2009.

The ARS already dedicates about EUR 10 million (USD 12.24 million) annually to the vector-control service or LAV, which is only a part of the overall budget being spent by municipalities, associations, and other actors to ensure vector control on the Island through various activities. The estimated population of the La Réunion Island in 2009 was about 816,360 inhabitants. ARS expenditure per person on vector control for 2009 was thus about USD 14.99/inhabitant (ARS expenditure does not represent total public expenditure on mosquito prevention and control – this is a value that we cannot capture, due to the role played by a number of different entities/authorities that in some way affect mosquito density on the Island). The annual GDP (Gross Domestic Product) per capita of La Réunion was EUR 17,884/inhabitant (USD 21,890/inhabitant) in 2009.

When compared to the expenditure of the vector-control service of the ARS, the total annual expenditure on protective measures for all households in La Réunion (i.e. USD 28.05 million) appears to be disproportionately high, a finding that has been pointed out by another study [28].

**Figure 4:**
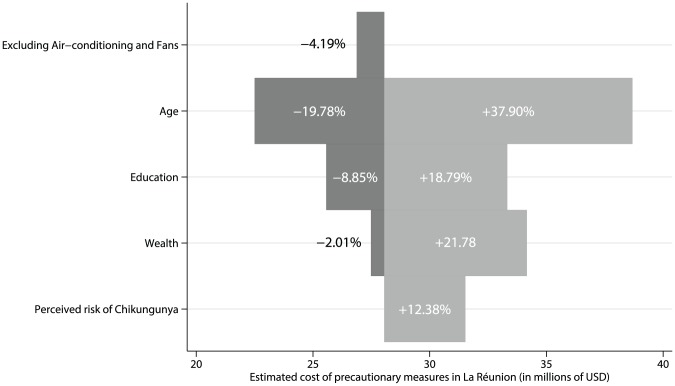
Tornado diagram of the change (in millions of Euro) in the estimated expenditure on protective measures related to variation in the main determinants of this expenditure. Lower sensitivity values are provided in dark grey. Higher sensitivity values are provided in light grey.

In order to test the sensitivity of our expenditure estimate to variation in the main factors influencing household-level expenditure, a univariate sensitivity analysis was carried out, as stated in the methods section. The factors influencing expenditure included perceived risk of a new epidemic of chikungunya, age, wealth, and education. We also tested the influence of excluding air-conditioning and fans from the expenditure calculation. The results are displayed graphically using a Tornado diagram in Figure 4. As guidance to interpret the findings in the diagram, for instance, age structure of the population has a large impact on estimated expenditure whereas variation in perceived risk of a new epidemic of chikungunya is unlikely to result in savings at the Island scale. The fact that the results are not strongly affected by the exclusion of air-conditioning and fans can be explained by the fact that the prices of these products were discounted in the main analysis.

## Discussion

To date, limited research has been performed on the relationship between objective or subjective measures of mosquito nuisance and their relationship with the use of personal measures at the household-level, despite the fact that these measures are an important method of protection against mosquito nuisance. Furthermore, no data currently exists on expenditure related to *Aedes* prevention and control in La Réunion at the household-level. This is surprising considering that participation of the community in vector-control programmes is understood to be critical in achieving sustainable and cost-effective control of mosquito density [29], [30], [31]. In recent years, new strategies (such as the sterile insect technique) aimed at controlling mosquito reproduction and ultimately the potential for this vector to transmit arboviruses, have emerged [32], [33], [34]. Such biological, chemical or genetically-oriented control programs are expensive and should be compared with the potential monetary savings at the household-level in the future.

The finding that household spending on protective measures against mosquitoes is related to zone-level vector density as measured by the Breteau index, but not to household-level measures of vector density is justifiable considering the role played by the socio-spatial environment on the risk of emerging infectious diseases such as chikungunya [35] and the findings of our study of a significant association between expenditure on protective measures and the perceived threat of another chikungunya outbreak on the Island. The situation varies for dengue, and the risk of an epidemic of this disease was not found to impact household expenditure on protective measures. The reason for this could be related to the way dengue is conceptualized by locals, which is different from their conceptualization of chikungunya. While it is evident that news of the dengue outbreak underway at the time of the study did influence risk perception, it is possible that this disease is not being linked to mosquitoes in La Réunion. In fact, members of the general public often think that dengue is synonymous with influenza, a mistaken notion that is perpetuated because both diseases are commonly referred to as “grippe” (or flu) on the Island. This confusion highlights the need for public health messages that are targeted to this audience and that specifically point out the role of the mosquito in the transmission of dengue, as well as the difference that exists between this disease and influenza. Improved education campaigns could be tested using this survey as a baseline for future randomized experiments.

The next concern related to public health is that household budgets on protective measures appear to be dedicated to measures that may not be the most effective in reducing *Aedes* nuisance. Most people interviewed in this study quoted mosquito coils as the most frequently used product against mosquitoes (Table 1), which is consistent with the findings of other studies conducted in urban areas [18], [21]. The effectiveness of mosquito coils is yet to be established with some official health recommendations for travelers judging them ineffective, while authors including Mulla et al [28] declare that they are relatively effective for reducing the frequency of mosquito bites. Overall, it seems that the effectiveness of coils may vary depending on the chemicals used in their manufacture and the conditions under which they are used [36], [37]. Mixed findings such as these highlight the need for studies dedicated to establishing the effectiveness of different protective measures. These studies could be the basis for introducing new interventions, such as the introduction of taxation on ineffective products, which could be used to promote the right behaviours and contribute to the financing of more effective vector-control programmes. This study cannot provide sufficient information to favour the introduction of such a taxation policy but it is recommended that public health authorities consider this as a potential option once findings of the varying effectiveness of different personal measures is substantiated through other studies.

### Limitations

The general limitations of this study are related to the fact that our expenditure estimates could be underestimated or overestimated. Being that the study was carried out in July which is a less favourable month for mosquitoes, it is probable that our household expenditure is under-estimated (conservative bias). Moreover, we have excluded from the study residents living in high-rise buildings, apartments and flats. This is an additional source of underestimation for our expenditure estimate. However, it is also possible that the expenditure estimates have been overestimated as participants may have reported using more protective measures than they actually do in reality and because the measures they use target mosquito nuisance in general and not only that created by *Aedes albopictus*, the focus of our study. In La Réunion the name “Tiger mosquito” is not commonly used and it is probable that the majority of people cannot distinguish between different species of mosquitoes. This is a fair assumption considering the low level of general knowledge on mosquitoes we found in this study sample. This assumption should be tested in future studies in La Réunion by specifically asking participants whether they can correctly identify *Aedes albopictus*, which has been found to be the case in other parts of the world, including the South of France [38]. Our inclusion criteria allowed us to include zones located in specific geographical settings (i.e. located near the coast (less than 500 metres in altitude) where the presence of mosquitoes from one year to the next is most likely to remain stable. This is another source for potential overestimation of household expenditure. Using these inclusion criteria could have resulted in a slight overestimation of the average Breteau index in our sample compared to the Island average. Indeed, in terms of risk indicators, the average Breteau index in our sample is 42.28 [37.92–46.63]. The estimate provided by the ARS in 2012 for the whole Island is 30.82 [27.19–34.44] and 38 [34.37–41.62] in 2011. Nevertheless, this slight overestimation of the Breteau Index should not have much influence on the estimates for household-level expenditure obtained from the regression analyses. This is because we have a variety of situations represented through the positive and negative zones used in this study.

Our household-level expenditure estimations may also be limited by the fact that we have no longitudinal survey to confirm usage patterns of products across time. In addition to this, using model predictions with average values of covariates can lead to over-estimation of expenditure when this is made on the scale of an Island.

In this article, we were particularly interested in expenditure on chemically-based protective measures or repellents (including insecticide-treated nets). We did not focus on ecological interventions (such as eliminating mosquito breeding sites and stagnant water) or other protective behaviours (such as limiting outdoor activities), information which would be valuable when measuring opportunity costs due to time spent on these activities or the impact of mosquito nuisance on quality of life. This could be the subject of another study in the future.

Another limitation is that this cross-sectional study cannot fully explain why people use some chemical measures that are judged to be ineffective by French public authorities. It is probable that habits or tradition continue to play a role in the use of certain measures to repel or kill mosquitoes in La Réunion, for example the use of fire. Identifying the reasons for these continuing behaviours and the use of measures that may actually prove to be ineffective requires a qualitative study approach that would capture more information than is possible through use of standard questionnaires and quantitative methods.

Lastly, a key lesson from this cross-sectional survey is that longer-term research should be undertaken in order to take into account seasonal variations in protective behaviours against mosquito nuisance and disease threats in order to provide more robust conclusions.

### Conclusion

Differences in mosquito control practices at the local level involve the interplay of place, scale and politics [39]. This study is one of the first attempts to quantify household-level expenditure on protective measures against mosquitoes, a very important step considering that community involvement is considered to be at the heart of vector-control strategies in La Réunion and elsewhere. More importantly, longer-term studies on this subject, as well as studies on the effectiveness of different products, can be instrumental in determining potential savings at the household-level due to improvements in public messages and the introduction of new policies or interventions that are currently considered as being too expensive. Finally, it is evident that household-level behaviour is differentially affected by subjective and objective measures of exposure to *Aedes albopictus*. Both variables need to be taken into account when explaining the use of chemically-based protective measures against mosquitoes and any related variations in expenditure.

## Supporting Information

Checklist S1 STROBE checklist.
(PDF)
